# From reactive trials to regional vaccine sovereignty: a pan-ebolavirus vaccine-readiness framework for East Africa’s Sudan virus and Bundibugyo virus threats

**DOI:** 10.3389/fpubh.2026.1890576

**Published:** 2026-08-05

**Authors:** Okechukwu Paul-Chima Ugwu, Chinyere Nneoma Ugwu, Godson Emeka Anyanwu, Mariam Basajja, Maryann Chiamaka Ebuoh

**Affiliations:** 1 Department of Research, Publication and Extension, Kampala International University, Kampala, Uganda; 2 Department of Anatomy, Faculty of Biomedical Sciences, Kampala International University, Kampala, Uganda; 3 Department of Human Anatomy, State University of Medical and Applied Sciences, Igbo Eno, Enugu, Nigeria

**Keywords:** Bundibugyo virus, East Africa, Ebola, outbreak preparedness, ring vaccination, Sudan virus disease, vaccine development, vaccine sovereignty

## Abstract

Vaccines have transformed preparedness for Zaire ebolavirus disease, but East Africa still faces recurrent risk from Sudan virus and Bundibugyo virus, for which licensed and routinely deployable vaccines remain unavailable. Uganda’s 2025 Sudan virus disease outbreak changed the field because it hosted the first clinical efficacy trial of a Sudan virus vaccine during an active outbreak. That was a major scientific achievement, yet it also exposed a more basic weakness: the region still relies too heavily on outbreak-triggered trial activation rather than on a standing preparedness architecture. We argue that East Africa now needs a pan-ebolavirus vaccine-readiness platform built around the threats it repeatedly faces, and we propose that such a platform should be embedded within the broader filovirus preparedness system rather than treated as a stand-alone vaccine project. Such a platform would link genomic surveillance, prototype vaccine libraries, harmonised ethics and regulatory pathways, adaptive ring-vaccination protocols, cold-chain corridors, community trust systems, African-led data stewardship, and manufacturing partnerships. We further argue that Sudan virus and Bundibugyo virus should no longer be treated as occasional vaccine gaps; they are regional preparedness priorities that also expose persistent therapeutic gaps. A sovereignty-oriented model would not replace global collaboration; it would make that collaboration more balanced by enabling East African institutions to lead trial readiness, deployment, evidence generation, and equitable access before the next spillover occurs.

## Introduction

Ebola vaccine development is no longer starting from an empty field. The rVSV-vectored vaccine programme changed expectations for what outbreak control can look like when scientific preparation, ethical approval, and deployment systems are already aligned. The Guinea ring-vaccination trial showed high protection, and later work in Liberia strengthened the evidence base for vaccine safety and field deployment (1, 2). What has not happened, though, is an equal spread of that progress across the ebolavirus genus.

East Africa remains exposed to Sudan virus and Bundibugyo virus. That risk is not abstract. Uganda has experienced repeated Sudan virus disease outbreaks, and the World Health Organization has described Sudan virus disease as enzootic in the region, with previous Ugandan outbreaks in 2000, 2011, two in 2012, and again in 2022 (3, 4). The 2022 outbreak alone caused 164 cases and 77 deaths across nine districts, a sharp reminder that these viruses are part of the region’s actual outbreak ecology rather than a distant contingency (4).

The 2025 Sudan virus disease outbreak in Uganda marked a turning point. Uganda’s Ministry of Health, the World Health Organization, and partners launched the first clinical efficacy trial of a Sudan virus vaccine during an active outbreak, using a ring-vaccination design and doing so within days of outbreak confirmation (5–7). That was a genuine advance. Still, in our view, the episode also made something else hard to ignore. If Sudan virus disease keeps returning to East Africa, preparedness cannot keep depending on the chance alignment of transmission, external mobilisation, and emergency trial start-up.

We use this Perspective to argue for a shift from reactive trials to regional vaccine sovereignty. By vaccine sovereignty, we do not mean isolation from international partners. We mean the ability of East African institutions to shape vaccine preparedness, trial activation, data stewardship, deployment, and longer-term access for the pathogens most relevant to the region. We also wish to be precise about scope from the outset. A fully pan-ebolavirus vaccine remains a long-term scientific aspiration, whereas a pan-ebolavirus preparedness system spanning diagnostics, vaccines, therapeutics, and outbreak research is already largely achievable. This distinction matters, and we return to it throughout.

## Methods

This article is a narrative evidence synthesis and policy perspective. It is not a systematic review, a meta-analysis, or a primary field study, and it should not be read as one. The literature search was undertaken in April and May 2026 using PubMed, Web of Science, and Google Scholar, alongside targeted searches of World Health Organization, IAVI, Sabin Vaccine Institute, and CEPI materials for outbreak-specific developments that had not yet fully entered the peer-reviewed literature. Search terms combined Sudan virus, Bundibugyo virus, Ebola vaccine, ring vaccination, Uganda, East Africa, vaccine manufacturing, clinical trial preparedness, genomic surveillance, and community engagement.

Priority was given to peer-reviewed studies, major outbreak notices from the World Health Organization, and programme documents from recognised vaccine developers or preparedness funders where those documents provided unique and time-sensitive information on the 2024 to 2026 Sudan virus and Bundibugyo virus vaccine landscape. Because the aim was to support a policy and advocacy argument rather than to estimate a pooled effect, the search was purposive rather than exhaustive: sources were selected for their relevance to the preparedness questions at hand, and no formal risk-of-bias appraisal or quantitative synthesis was performed. The final selection was used to support the perspective and its policy recommendations.

*A note on terminology.* For consistency, we use “Sudan virus” and “Bundibugyo virus” for the viruses, “Sudan virus disease” and “Bundibugyo virus disease” for the clinical conditions they cause, and “Zaire ebolavirus” only where the formal species designation is required. “Ebolavirus” is reserved for genus-level statements. These conventions are applied throughout.

### From reactive trials to regional vaccine sovereignty

The current Ebola vaccine model is still largely reactive. Candidate vaccines tend to move to the front of the queue when an outbreak creates urgency, and the operational value of the platform often depends on whether doses, cold-chain systems, trial protocols, trained teams, and ethics approvals were already in place. Uganda’s 2025 Sudan virus disease trial illustrates both the promise and the weakness of this model. The World Health Organization reported that candidate doses had been pre-positioned in Kampala, that protocols had already been developed through collaborative preparedness work, and that the ring-vaccination trial was launched only 4 days after the outbreak was confirmed (6–8). IAVI likewise reported that the candidate vaccine had already been positioned in the country and that this readiness was central to rapid trial activation (9).

The speed was impressive, but the model remains fragile. Outbreaks can be short, geographically dispersed, politically sensitive, or interrupted by cross-border movement. When transmission declines quickly, the window for generating convincing efficacy data narrows. Uganda’s 2025 outbreak ended with 14 total cases and four deaths, which was a public health success, although it also underscored how difficult it can be to generate robust efficacy evidence during small outbreaks (7, 9). A reactive model, in other words, may achieve rapid mobilisation without guaranteeing durable evidence.

We propose that a sovereignty-oriented model should start earlier. It asks whether the region already has the scientific and institutional machinery to select, test, regulate, deploy, and evaluate a vaccine before transmission intensifies. That means standing trial protocols, pre-agreed ethics pathways, trained field teams, mapped cold-chain routes, community advisory mechanisms, sequencing capacity, and clear agreements on data ownership and benefit sharing. In such a system, East African countries are not merely sites where emergency studies happen; they are co-designers and co-owners of the preparedness architecture.

### Why Sudan virus and Bundibugyo virus matter for East Africa

Sudan virus disease is not a rare curiosity in Uganda; it is a recurrent regional threat. The 2025 trial was encouraging because it showed that Sudan virus-specific vaccine research can move from preparedness talk to operational action. The World Health Organization described the trial as the first ever clinical efficacy study of a Sudan virus vaccine during an outbreak, while the Sabin Vaccine Institute had already begun a Phase 2 trial of a separate Sudan virus candidate in Uganda and Kenya in 2024 (6, 10). At the same time, early clinical data from a bivalent ChAdOx1 Ebola virus and Sudan virus vaccine suggested that broader orthoebolavirus vaccine strategies are technically plausible, even if they are not yet field-ready for outbreak deployment (11).

Bundibugyo virus is arguably the sharper test of vaccine equity and preparedness commitment. It has caused severe outbreaks, yet it has not attracted the same sustained investment as Zaire ebolavirus or, more recently, Sudan virus. In May 2026, the World Health Organization confirmed a Bundibugyo virus outbreak affecting the Democratic Republic of the Congo and Uganda and stated that no licensed vaccine or specific therapeutic existed for Bundibugyo virus disease (12). CEPI reported at the same time that no Bundibugyo virus vaccine candidate had entered Phase I clinical trials, even though several candidates were in preclinical development (13).

Why has Bundibugyo virus vaccine development lagged so far behind? We suggest that the explanation is partly biological and partly structural. Biologically, the Bundibugyo virus glycoprotein is markedly divergent from that of Zaire ebolavirus. Across the surface glycoprotein, amino-acid identity between Ebola virus and Bundibugyo virus is only about 65%, and between Ebola virus and Sudan virus only about 55% (14). Because licensed vaccines and the recommended monoclonal-antibody therapies were developed against the Zaire glycoprotein, this divergence means that cross-protection cannot be assumed: the glycoprotein epitopes those products target differ at precisely the sites that matter for neutralisation, and conservation of the primary sequence does not reliably predict antibody cross-reactivity (14). This is why broadly protective approaches that target the more conserved internal fusion loop or glycoprotein stalk rather than the variable mucin-like and glycan-cap regions are scientifically attractive but technically demanding (15). Structurally, Bundibugyo virus has caused fewer and smaller recorded outbreaks than Zaire or Sudan ebolaviruses, so commercial and even philanthropic incentives have been weaker, and candidate products have rarely had an outbreak in which to be tested. The result is a self-reinforcing cycle in which low perceived demand slows development, and slow development leaves each new outbreak without ready tools.

That gap matters. If the region waits for every Bundibugyo virus outbreak before asking whether tools are ready, the response will remain late by design. We therefore argue that East Africa should treat Bundibugyo virus as a preparedness priority now, not as a side issue to revisit once the next outbreak is already under way (Table 1).

**Table 1:** East African pan-ebolavirus vaccine-readiness priorities.

Preparedness domain	Current gap	Sovereignty opportunity	Translational priority and evidence base
Genomic surveillance	Species confirmation and cross-border situational awareness can be delayed. Remote districts and fast-moving outbreaks are hardest to track.	Linked sequencing guides candidate selection in real time. Supports outbreak mapping and regional risk assessment.	Establish connected filovirus genomic nodes across national public health institutes and border laboratories (7, 12).
Clinical trial activation	Start-up depends on emergency mobilisation and outbreak size. Small outbreaks limit efficacy-evidence generation.	Standing adaptive and ring-vaccination protocols shorten activation time. Preserve scientific opportunity during small outbreaks.	Pre-approve core protocols for high-risk districts and maintain trained rapid-deployment trial teams (5–7).
Ethics and regulation	Sequential country-level review slows multi-country research. Shared scientific questions are reviewed in isolation.	Regional reliance pathways support faster review. Standards are maintained, not lowered.	Develop East African emergency ethics and regulatory harmonisation mechanisms for filovirus trials through AMA and AVAREF (6, 21).
Vaccine access	Candidate doses may be externally controlled. Doses can arrive too late for outbreak-linked evaluation.	Pre-positioned Sudan candidates speed deployment. Agreed access frameworks for Bundibugyo candidates.	Maintain transparent regional stockpile and access agreements for investigational products (8, 9, 13).
Cold chain and logistics	Remote districts are hard to serve quickly. High-mobility border corridors strain delivery.	Mapped cold-chain corridors connect outbreak zones, laboratories, and trial sites. More reliable delivery under pressure.	Stress-test vaccine delivery routes during inter-epidemic periods, including border crossings and referral networks (7).
Community trust systems	Engagement that begins after cases are confirmed breeds mistrust. Emergency research may be misunderstood.	Continuous engagement improves legitimacy, consent quality, and uptake. Trust is built before, not during, crises.	Work with survivor groups, health workers, religious leaders, school networks, and border communities between outbreaks (7, 18, 19).
Manufacturing partnerships	Regional access depends on external production and donation. Rare-filovirus demand is intermittent and hard to sustain.	Dual-purpose African fill-finish and technology-transfer partnerships. Stronger long-term access and bargaining power.	Link filovirus trials to technology-transfer arrangements and regional procurement, leveraging facilities such as Institut Pasteur de Dakar and Biovac (20, 21).
Data governance	Outbreak data may leave the region without clear rules. Ownership, authorship, and benefit sharing are unsettled.	African-led stewardship improves equity. Strengthens regional scientific leadership.	Agree transparent data-sharing and benefit-sharing rules before emergencies occur (17, 21).

The table is organised around translational functions rather than around isolated technical interventions. Each domain is grounded in evidence discussed in the main text. AMA = African Medicines Agency; AVAREF = African Vaccine Regulatory Forum.

### A pan-ebolavirus vaccine-readiness platform within a wider preparedness system

Before describing the platform, we want to draw one distinction clearly, because it shapes everything that follows. Ebolavirus vaccine development is a component of, but not the same as, ebolavirus preparedness. A preparedness system spans diagnostics, vaccines, therapeutics, and outbreak research, and much of that system is achievable in the near term. A single universal pan-ebolavirus vaccine is not. We therefore use “pan-ebolavirus readiness platform” to mean the institutional and logistical machinery that can be matched to whichever species is causing an outbreak, not a claim that one vaccine will cover the genus. Keeping vaccines, therapeutics, and diagnostics within a shared platform also helps ensure that the well-documented therapeutic gap for Sudan virus and Bundibugyo virus is not overlooked: as of the 2026 outbreak, no monoclonal-antibody therapy was licensed for either virus, and the pan-ebolavirus antibody cocktails furthest along in development, such as MBP134, remained investigational (14).

With that scope established, we propose that such a platform should combine eight linked functions. First, genomic surveillance needs to confirm virus species quickly and support cross-border situational awareness. Second, prototype vaccine libraries should keep Sudan virus and Bundibugyo virus candidates scientifically active between outbreaks rather than allowing development to stall. Third, harmonised ethics review and regional regulatory reliance can reduce delays that add little scientific value. Fourth, adaptive ring-vaccination protocols should remain ready for activation, alongside health-worker and geographically targeted designs where epidemiology warrants them. Fifth, cold-chain corridors must be mapped and stress-tested. Sixth, community trust systems should be built before emergencies. Seventh, regional data governance needs to protect African ownership of outbreak data and downstream scientific benefit. Eighth, manufacturing partnerships should link preparedness to fill-finish, technology transfer, and eventual production capacity within Africa.

The scientific platform also has to stay technologically flexible. Viral-vectored approaches remain central, especially for fast outbreak use, but they should not be the only route. A recent macaque challenge study found that a single-dose vesicular stomatitis virus Sudan virus vaccine could confer rapid protection, which supports the continued relevance of fast-acting species-specific platforms for ring-vaccination use (16). At the same time, adjuvanted multivalent glycoprotein approaches have shown broad antibody and T-cell responses across Ebola, Sudan, and Bundibugyo viruses, suggesting that broader protective strategies deserve sustained investment (15). The first-in-human ChAdOx1 bivalent Ebola virus and Sudan virus vaccine trial showed acceptable safety and immunogenicity, which adds to the case for multi-species platform development rather than species-by-species improvisation (11). To help readers locate these candidates against the proposed ideal platform, Table 2 summarises the current clinical pipeline for the three filoviruses discussed.

**Table 2:** Current clinical-stage vaccine pipeline for the three East African ebolaviruses.

Ebolavirus species	Most advanced clinical stage	Representative candidates and platforms	Principal remaining gap
Zaire ebolavirus	Licensed	rVSV-ZEBOV (Ervebo) and Ad26.ZEBOV/MVA-BN-Filo two-dose regimen; licensed and WHO-prequalified.	Reference platform; main gap is extending protection across species.
Sudan virus	Phase 2 and outbreak efficacy	ChAdOx1 biEBOV (bivalent EBOV–SUDV, first-in-human Phase 1); Sabin ChAd3-SUDV (Phase 2, Uganda and Kenya); ring-vaccination efficacy trial during the 2025 outbreak.	No licensed product; small outbreaks limit efficacy data; correlates of protection unconfirmed.
Bundibugyo virus	Preclinical only	Multivalent adjuvanted glycoprotein candidates and pan-ebolavirus approaches in preclinical development; no candidate in Phase 1 as of 2026.	No clinical-stage candidate; divergent glycoprotein limits cross-protection; weak commercial incentive.

Pipeline status is summarised from sources cited in the main text (6, 10–13, 15). EBOV = Ebola (Zaire) virus; SUDV = Sudan virus. The contrast across rows illustrates the uneven readiness that the proposed platform is intended to address.

### Regional systems, legitimacy, and implementation

A vaccine platform is not only a laboratory problem. Outbreak vaccination depends on trial capacity, logistics, and public legitimacy. Uganda already has relevant infrastructure on which a regional platform could build. Naluyima et al. (17) described how the Makerere University Walter Reed Project contributed to vaccine trial readiness in Uganda through clinical, laboratory, data-management, and community-engagement systems. That institutional base matters, because emergency research cannot be assembled from scratch each time a filovirus resurfaces.

Community legitimacy is just as central. Ring vaccination sounds elegant on paper, but it relies on contact tracing, rapid communication, consent, and trust. Soke et al. (18) argued that continuous community engagement is needed between outbreaks if adherence to Ebola response activities is to improve during crises, and Musoke et al. (19) documented the specific barriers that arise when engagement is left until cases are already confirmed. The World Health Organization’s report on Uganda’s 2025 Sudan virus disease outbreak points in the same direction: it noted that integrated risk communication and community engagement, including work with religious leaders, schoolteachers, traditional healers, and other local influencers, helped improve access, trust, and cooperation during the response (7). A readiness platform that treats trust as an afterthought will not work when the pressure rises.

There is also a manufacturing and governance dimension, and it deserves a candid feasibility assessment rather than rhetoric about self-reliance. Africa still produces only about 1% of the vaccines it uses, and the continent’s current ecosystem continues to face constraints in financing, regulatory maturity, the distribution of clinical trials, and sustainable manufacturing demand (20, 21). For rare filoviruses these constraints are acute: outbreak-only demand is intermittent and difficult to forecast, so a dedicated Sudan virus or Bundibugyo virus production line would sit idle between outbreaks and would be hard to sustain on commercial logic alone. We therefore do not advocate immediate, full-scale local manufacturing of rare filovirus vaccines. The more realistic near-term pathway is to dual-purpose existing and emerging African facilities through fill-finish partnerships and technology transfer, reserving filovirus capability as a surge option within multi-product platforms.

Such facilities already exist or are coming online. The Institut Pasteur de Dakar, the only WHO-prequalified vaccine manufacturer in Africa, has commissioned the MADIBA fill-finish facility, designed for live viral, mRNA, and protein-subunit vaccines, and is the CEPI preferred African manufacturer slated to receive a Lassa fever vaccine candidate for production (22). Biovac in South Africa has moved from fill-finish toward partial upstream production and mRNA research, and BioNTech’s modular mRNA facility in Kigali positions Rwanda as a regional node (20). Within East Africa, Uganda’s Dei Biopharma has initiated domestic manufacturing ambitions. None of these is yet a filovirus producer, but their flexible, multi-platform design is precisely what makes a surge-capable, dual-purpose model plausible. For East Africa, then, vaccine sovereignty should be framed not as building filovirus plants from scratch but as securing pre-negotiated fill-finish, technology-transfer, and procurement agreements that can be activated when a regional candidate is ready.

Regulatory coordination is the connective tissue for all of this. Within the region, Uganda’s National Drug Authority, Kenya’s Pharmacy and Poisons Board, Tanzania’s Medicines and Medical Devices Authority, and equivalent bodies in the Democratic Republic of the Congo and other affected nations each hold relevant expertise but currently review largely in sequence, which slows multi-country research even when the scientific question is shared. Formalising regional reliance mechanisms in which one authority’s assessment can be recognised by others can build on existing relationships while maintaining scientific standards. At continental level, the African Medicines Agency provides a centralising regulatory platform intended to harmonise national requirements and reduce the cost and time of navigating many separate regimes, while the African Vaccine Regulatory Forum offers a mechanism for joint review of vaccine trial applications. Together these bodies could support harmonised, reliance-based review tailored to filovirus vaccine trials, reducing redundant delays without compromising safety or efficacy assessment (21).

### Research and policy priorities

Several priorities follow from this framework. One is scientific. Sudan virus and Bundibugyo virus vaccine development still need stronger evidence on correlates of protection, durability, and cross-species immunogenicity, and the marked glycoprotein divergence of Bundibugyo virus makes this evidence especially important (11, 14–16). Without those data, licensure is likely to remain heavily dependent on outbreak-based efficacy trials, which are valuable but unpredictable.

Another priority is operational. Candidate doses, ring-vaccination protocols, laboratory plans, and ethics approvals need to be ready before outbreaks, not built during them. Uganda’s 2025 experience shows what is possible when candidate doses are pre-positioned and protocols are already prepared, yet it also shows how much still depends on a small number of institutions and external coordination (5, 6, 8, 9). We propose that distributed preparedness networks spanning multiple sites across Uganda, Kenya, the Democratic Republic of the Congo, and other at-risk countries would reduce this dependence on single-site readiness.

A third priority is political and financial. Bundibugyo virus vaccine development is unlikely to move quickly on commercial logic alone. Public and philanthropic financing, regional coordination, and explicit portfolio decisions will be needed if East Africa is to avoid a cycle in which the least commercially visible virus remains the least prepared for. CEPI’s 2026 Bundibugyo virus statement, together with the (12) outbreak notice, makes clear that the current pipeline is still too thin for that virus (12, 13).

Fourth priority concerns therapeutics, and we flag it deliberately so that vaccines are not pursued in isolation. The same glycoprotein divergence that complicates Bundibugyo virus vaccines also limits the Zaire-specific monoclonal antibodies, leaving both Sudan virus and Bundibugyo virus disease without a licensed specific treatment as of 2026 (12, 14). A preparedness platform that advanced vaccines while ignoring this therapeutic gap would be incomplete. Cross-protective antibody cocktails in development should therefore be carried alongside vaccine candidates within the same readiness architecture.

Finally, manufacturing translation needs to be tied to trial participation. Africa’s wider vaccine ecosystem is advancing, but substantial gaps remain in regulation, financing, and sustained industrial readiness (21). We propose that a regional pan-ebolavirus strategy should connect trials to fill-finish capacity, technology transfer, and procurement commitments rather than leaving manufacturing as an afterthought once efficacy data become available.

**Figure 1:**
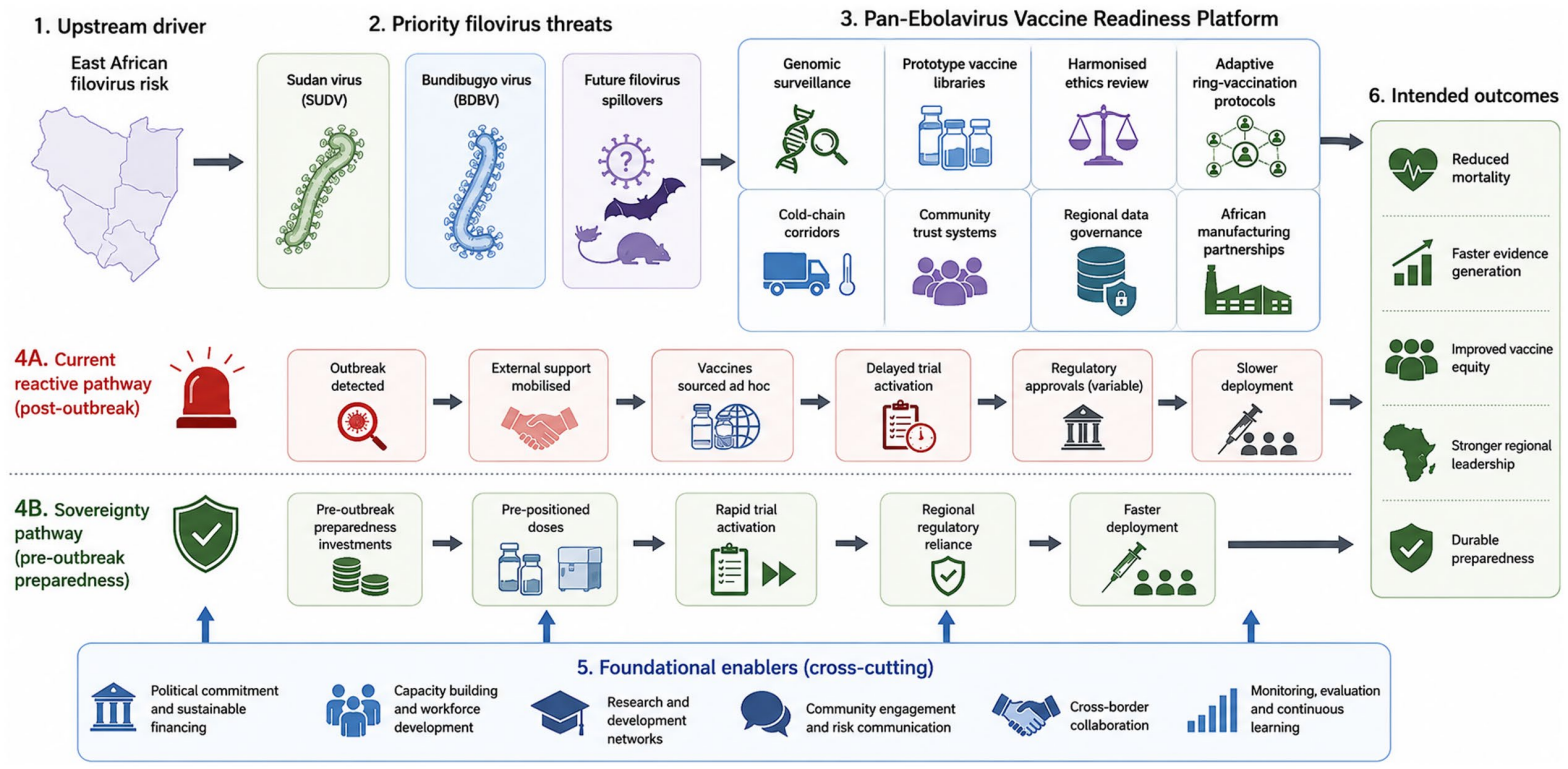
Conceptual framework for East African pan-ebolavirus vaccine sovereignty.

East African filovirus risk is positioned as the upstream driver of three priority threats: Sudan virus, Bundibugyo virus, and future filovirus spillovers. These converge on a Pan-Ebolavirus Vaccine Readiness Platform comprising genomic surveillance, prototype vaccine libraries, harmonised ethics review, adaptive ring-vaccination protocols, cold-chain corridors, community trust systems, regional data governance, and African manufacturing partnerships. The figure contrasts the present reactive pathway with a sovereignty pathway built on pre-outbreak preparedness, pre-positioned doses, rapid trial activation, regional regulatory reliance, and faster deployment. Intended outcomes are reduced mortality, faster evidence generation, improved vaccine equity, stronger regional leadership, and durable preparedness (Figure 1).

## Discussion

Uganda’s 2025 Sudan virus disease vaccine trial should be recognised for what it was: a landmark demonstration that rapid clinical evaluation is possible during an outbreak when preparation has happened in advance (5–7). But it should not be mistaken for a finished model. In a way, its biggest lesson may be structural. Speed came from preparedness work that had already been done, from pre-positioned doses to previously developed protocols and trained teams (8, 9).

That is why the core question is no longer whether East Africa can host a filovirus vaccine trial. It clearly can. The sharper question is whether the region will continue to depend on emergency-triggered activation for pathogens it already knows it faces, or whether it will build a standing platform able to govern its own priorities. For Sudan virus, that means moving from episodic response to sustained readiness. For Bundibugyo virus, it means refusing to let neglect masquerade as rarity.

We also want to be candid about the barriers, because an advocacy argument that ignores them is weak. Three stand out. The first is demand and financing: rare-filovirus products generate intermittent, hard-to-forecast demand, so sustaining manufacturing and stockpiles will require pooled, largely public or philanthropic financing rather than market signals. The second is scientific: the glycoprotein divergence of Bundibugyo virus, the unresolved correlates of protection, and the difficulty of generating efficacy data during small outbreaks together mean that licensure pathways for these viruses remain genuinely uncertain. The third is institutional: regional regulatory reliance and African-led data governance depend on political will and on harmonisation mechanisms, such as the African Medicines Agency, that are still maturing. None of these barriers is trivial, and none is a reason for inaction; rather, each defines where investment and coordination should be concentrated.

The policy implications follow directly. Ministries of health and their partners should treat Sudan virus and Bundibugyo virus as standing regional priorities with named budget lines, pre-negotiated trial and regulatory pathways, and explicit manufacturing and procurement agreements, rather than as emergencies to be improvised when the next outbreak begins. Crucially, vaccines should be advanced inside a broader preparedness system that also carries diagnostics and therapeutics, so that the documented treatment gap for these viruses is closed in parallel with the vaccine gap.

## Conclusion

East Africa has the outbreak experience, institutional partners, and scientific capability needed to lead the next phase of filovirus vaccine preparedness. What is still missing is a permanent platform that treats Sudan virus and Bundibugyo virus as predictable regional threats rather than exceptional emergencies. We have argued that a pan-ebolavirus vaccine-readiness platform, embedded within a wider preparedness system spanning diagnostics, vaccines, and therapeutics, would not solve every scientific question overnight, but it would shorten the interval between outbreak detection, species confirmation, candidate selection, ethical approval, community engagement, deployment, and evidence generation. That shift is overdue. The future of filovirus vaccine development in East Africa should depend less on how quickly external actors can mobilise after spillover and more on how well regional systems are prepared before it begins.

## Data Availability

The original contributions presented in the study are included in the article/supplementary material, further inquiries can be directed to the corresponding author.
